# Supplementary figures and images for: Neuronal Avalanches in Input and Associative Layers of Auditory Cortex
Source: Front Syst Neurosci. 2019 Sep 4;13:45. doi: 10.3389/fnsys.2019.00045 (PMC6737089; doi:10.3389/fnsys.2019.00045)

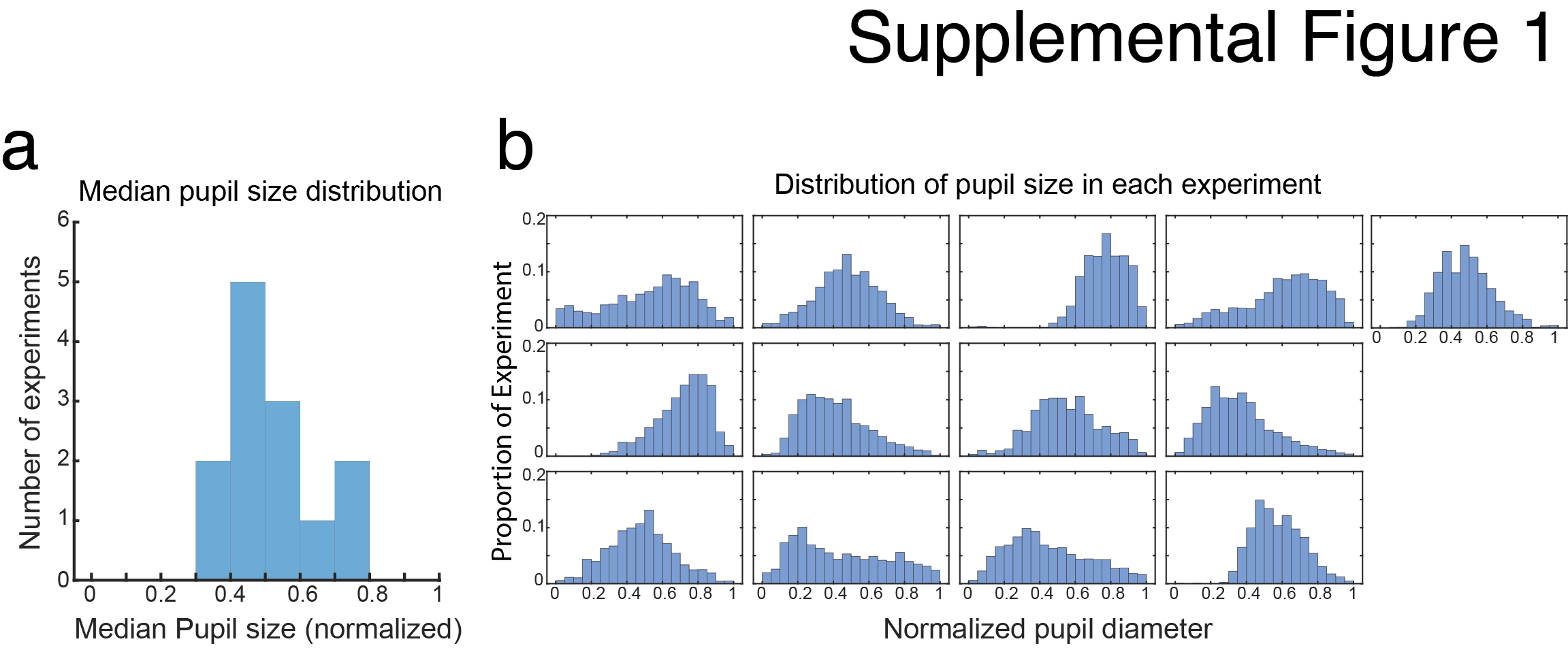

Supplement: FIGURE S1 — Mouse pupil diameter indicates an intermediate arousal state on average.(a) Histogram of median pupil diameter computed from each experiment. (b) Histogram of pupil diameters over the course of each experiment where each subplot represents an individual imaging session. Most experiments exhibit a peak near the middle of the pupil diameter range, indicating an intermediate arousal state. [file Image_1.png]

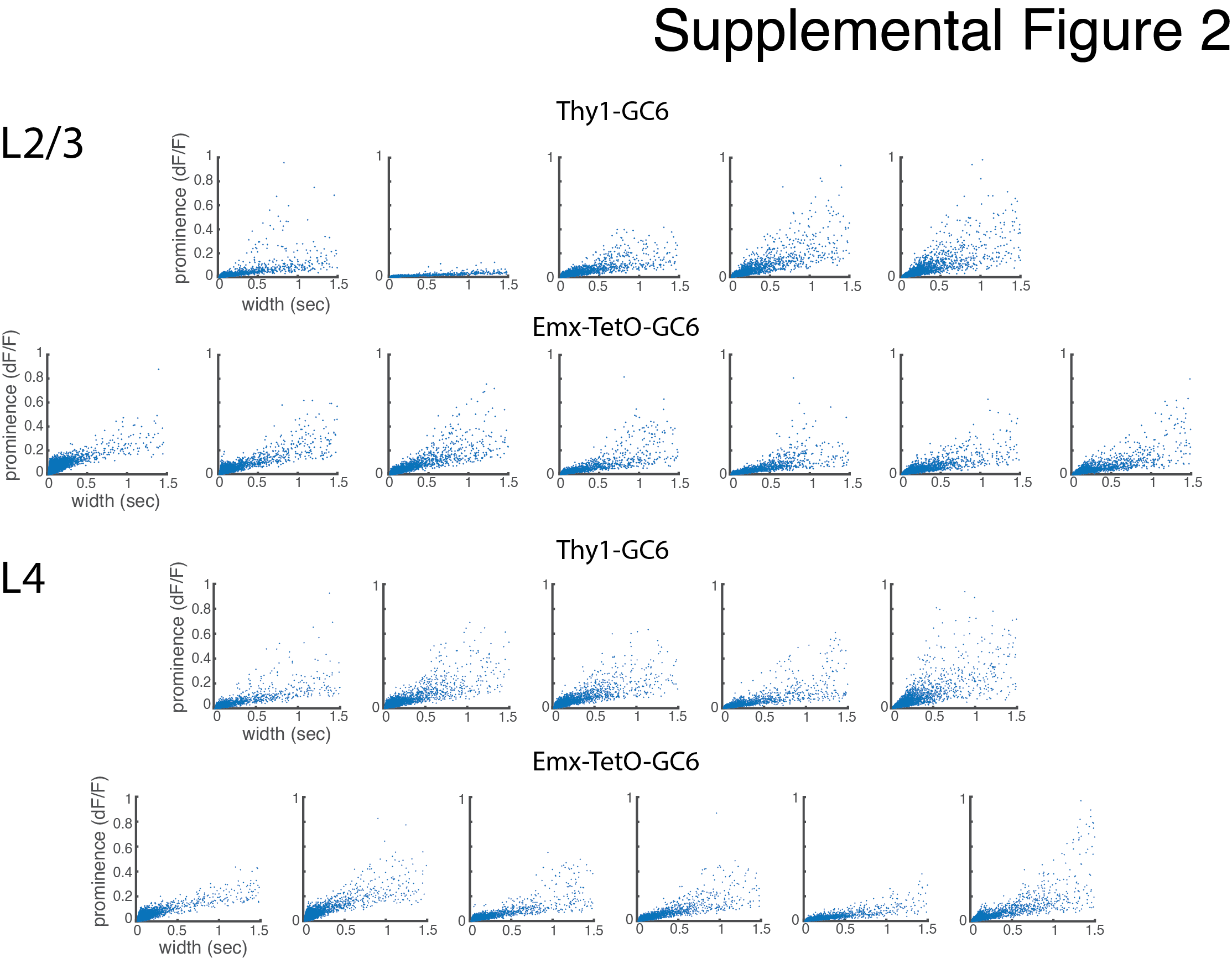

Supplement: FIGURE S2 — No epileptiform activity evident in any mouse strains. Scatter plots of prominence and width of peaks taken from the mean fluorescence trace over time of the entire 2-photon field of view. Each plot represents an individual experiment. Epileptiform activity would be evident as a secondary cluster of points at high prominence and low width. [file Image_2.png]

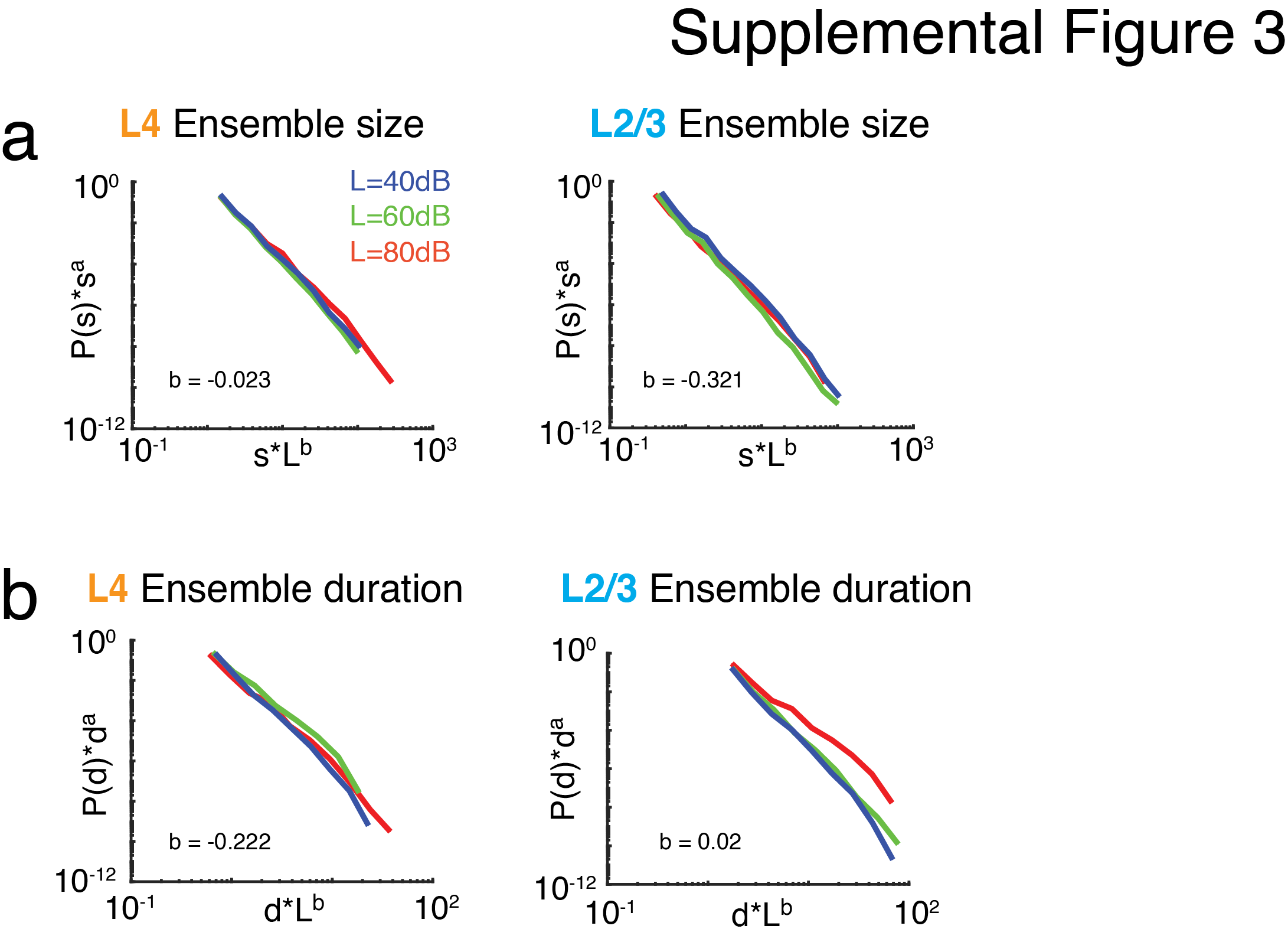

Supplement: FIGURE S3 — Power laws are independent of stimulus sound level. (a) Re-scaled power law distributions for ensemble sizes from L4 (left) and L2/3 (right) data. Variable s indicates ensemble size, P(s) indicates probability of ensemble size s, a is the best fit slope from the original ensemble distributions, L is the stimulus sound level, and b is the scaling factor. (b) Conventions as in a, except for ensemble duration where variable d is ensemble duration. [file Image_3.png]
