# Supplementary material for: Neuronal Avalanches in Input and Associative Layers of Auditory Cortex
Source: Front Syst Neurosci. 2019 Sep 4;13:45. doi: 10.3389/fnsys.2019.00045 (PMC6737089; doi:10.3389/fnsys.2019.00045)
Supplement: TABLE S1 — Statistical comparisons of the log-likelihood ratio values of avalanche size distributions and shuffled avalanche size distributions. [file Table_1.docx]

|  | L2/3  80 dB | L2/3  60 dB | L2/3  40 dB | L4  80 dB | L4  60 dB | L4  40 dB |
| --- | --- | --- | --- | --- | --- | --- |
| LLR Actual | 2573.9 | 2448.7 | 2094.4 | 1357.4 | 1480.3 | 1213.9 |
| Mean shuff | -0.3 | 14.2 | 3.6 | 104.3 | 99.8 | 115.5 |
| std shuff | 40.1 | 50.6 | 47.1 | 39.3 | 35.3 | 39.5 |
| Test | 't-test' | 't-test' | 't-test' | 't-test' | 't-test' | 't-test' |
| P-Value | 5.90 x10^-181^ | 1.40 x10^-168^ | 3.66 x10^-165^ | 7.05 x10^-151^ | 1.19 x10^-159^ | 4.52 x10^-145^ |

**Supplemental Table 1.** Statistical comparisons of the log-likelihood ratio values of avalanche size distributions and shuffled avalanche size distributions.
