# Supplementary material for: Neuronal Avalanches in Input and Associative Layers of Auditory Cortex
Source: Front Syst Neurosci. 2019 Sep 4;13:45. doi: 10.3389/fnsys.2019.00045 (PMC6737089; doi:10.3389/fnsys.2019.00045)
Supplement: TABLE S2 — Statistical comparisons of the log-likelihood ratio values from avalanche duration distributions and shuffled avalanche duration distributions. [file Table_2.docx]

|  | L2/3  80 dB | L2/3  60 dB | L2/3  40 dB | L4  80 dB | L4  60 dB | L4  40 dB |
| --- | --- | --- | --- | --- | --- | --- |
| LLR Actual | 1361.4 | 1337.9 | 1246.8 | 824.0 | 946.8 | 817.5 |
| Mean shuff | 356.8 | 381.0 | 360.9 | 422.9 | 423.8 | 433.2 |
| std shuff | 36.6 | 41.5 | 42.0 | 35.3 | 36.6 | 35.9 |
| Test | 't-test' | 't-test' | 't-test' | 't-test' | 't-test' | 't-test' |
| P-Value | 1.56 x10^-144^ | 4.72 x10^-137^ | 3.25 x10^-133^ | 9.89 x10^-107^ | 1.75 x10^-116^ | 3.30 x10^-104^ |

**Supplemental Table 2.** Statistical comparisons of the log-likelihood ratio values from avalanche duration distributions and shuffled avalanche duration distributions.
